# Growth Trajectories in Infants From Families With Plant-Based or Omnivorous Dietary Patterns

**DOI:** 10.1001/jamanetworkopen.2025.57798

**Published:** 2026-02-05

**Authors:** Kerem Avital, Naomi Fliss-Isakov, Danit R. Shahar, Moran Blaychfeld-Magnazi, Sivan Ben-Avraham, Sigal Tepper, Uri Hamiel

**Affiliations:** 1 Department of Epidemiology, Biostatistics, and Community Health Sciences, Ben Gurion University of the Negev, Be’er Sheva, Israel; 2 Israel Ministry of Health, Jerusalem, Israel; 3 Gertner Institute for Epidemiology and Health Policy Research, Sheba Medical Center, Tel Hashomer, Israel; 4 Department of Health Promotion, School of Public Health, Gray Faculty of Medical and Health Sciences, Tel Aviv University, Tel Aviv, Israel; 5 Department of Nutrition Sciences, Ariel University, Ariel, Israel; 6 Department of Nutritional Sciences, Tel-Hai College, Tel Hai, Israel; 7 Genetics and Genomics Institute, Tel Aviv Sourasky Medical Center, Tel Aviv, Israel; 8 Gray Faculty of Medical and Health Sciences, Tel Aviv University, Tel Aviv, Israel

## Abstract

**Importance:**

The safety of plant-based family diets, particularly vegan diets, during pregnancy and infancy is debated. Large population data on infant growth are scarce.

**Objective:**

To examine whether family dietary patterns (vegan, vegetarian, and omnivorous) are associated with growth trajectories, weight, and length among infants.

**Design, Setting, and Participants:**

This retrospective cohort study used data collected from January 1, 2014, through December 31, 2023, from a national network of public family care centers in Israel providing health and developmental surveillance for infants. Singleton births of infants gestational age 32 weeks or later without congenital malformations or birth weight less than 1500 g were followed up for 24 months. The data were analyzed between November 17, 2024, and December 6, 2025.

**Exposure:**

The family diet as recorded at least 6 months after delivery.

**Main Outcomes and Measures:**

The primary outcome was infant length. Secondary outcomes included weight, head circumference, stunting (length-for-age *z* score less than –2), underweight (weight-for-length *z* score less than –2), and overweight (weight-for-length *z* score >2). Growth trajectories were analyzed using linear mixed-effects models, and nutritional status at birth, early infancy (ie, first 60 days of life), and 24 months were analyzed using logistic regression.

**Results:**

Among 1 198 818 infants (mean [SD] gestational age, 39.2 [1.5] weeks; 53.2% male), 98.5% were from omnivorous households; 0.3% from vegan households, and 1.2% from vegetarian households. Differences in early-infancy length and length-for-age *z* scores among dietary groups were small (World Health Organization *z* score ≤0.3), and stunting prevalence was similar across groups (from 7.0% in the vegan and vegetarian groups to 7.1% in the omnivorous group), while underweight was more common in infants in the vegan vs omnivorous groups (adjusted odds ratio, 1.37 [95% CI, 1.15-1.63]). By age 24 months, stunting prevalence declined to 3.1%, 3.4%, and 3.9% in omnivore, vegetarian, and vegan groups, respectively, with no significant differences among the groups. Underweight and overweight were also low, with no differences by dietary group at age 24 months. Mean differences for weight, length, and head circumference were clinically minor (World Health Organization *z* score <0.2) and diminished further in adjusted longitudinal models.

**Conclusions and Relevance:**

In this cohort study, infants from vegan households had growth patterns similar to those from omnivorous households, with a higher odds of early underweight that decreased by age 24 months. In the context of developed countries, these findings seem reassuring. Further research should examine vegan diet quality and the impact of nutritional counseling during pregnancy and infancy in supporting optimal infant development.

## Key Points

**Question:**

What is the association between family dietary patterns (vegan, vegetarian, omnivorous) and infant growth trajectories, weight status, and stunting in early childhood?

**Findings:**

This cohort study in 1 198 818 infants revealed that infants from vegan households exhibited minimal differences in mean growth compared with their counterparts from omnivorous households. However, infants from vegan households had a modestly higher odds of underweight in early infancy, although these differences diminished by age 24 months.

**Meaning:**

These findings suggest that family vegan dietary patterns may support appropriate infant growth, but further work is needed to clarify how vegan diet quality and nutritional counseling during pregnancy and infancy support optimal infant development.

## Teaser

This cohort study examines the association of family dietary patterns (vegan, vegetarian, and omnivorous) with growth trajectories, weight, and length among infants in Israel.

## Introduction

The first 1000 days of life, including gestation and the first 2 years, constitute a crucial period for human development.^1^ The mother’s and child’s nutritional statuses during this period have substantial short-term health implications.^2^ Early-life nutrition affects the growth risk of developing noncommunicable diseases^2^ and obesity^3^ and may influence brain development.^4^ During this period, growth-related health concerns for children range from stunting and underweight to obesity.

Plant-based diets have been increasingly adopted in Western countries.^5^ Evidence suggests that vegan nutrition may reduce the risk of noncommunicable diseases^5^ and improve cardiometabolic outcomes.^6^ Nevertheless, concerns persist about nutritional adequacy in pregnancy and early childhood, particularly with regard to vitamin B_12_, iron, iodine, vitamin D, calcium, and long-chain omega-3 fatty acids.^7^ However, evidence on how vegan or vegetarian family dietary patterns influence early-life growth remains limited.^8,9^ No nationwide study has compared growth trajectories and growth status indicators among infants from vegan, vegetarian, and omnivorous households. To address this lack of data, we analyzed the association between household dietary patterns and longitudinal growth patterns and weight status of a large cohort of Israeli infants.

## Methods

### Study Setting and Data Source

This cohort study was a nationwide, retrospective study of singleton children born in Israel between January 1, 2014, and December 31, 2023. Data were sourced from TIMNA, the Israeli Ministry of Health’s (MOH’s) national research platform for large-scale research using deidentified health records. This study was approved by the MOH Research Ethics Board, with a waiver of informed consent because the study used deidentified, retrospective data and posed minimal risk to participants. The study adhered to the Strengthening the Reporting of Observational Studies in Epidemiology (STROBE) reporting guideline.

Anthropometric data (weight, length, and head circumference) and breastfeeding status were extracted from the MOH Family Child Center (FCC) database, which compiles records from MOH and municipality-operated clinics delivering free universal preventive care. Data from health maintenance organization–operated centers were not included; consequently, coverage represents approximately 70% of captured data rather than the total available service access. Follow-up included anthropometric evaluations, developmental assessments, screening tests, and vaccination records for infants from birth to age 6 years. To minimize selection bias due to differential follow-up, we restricted analyses to visits conducted before age 30 months, when attendance was highest owing to the routine vaccination schedule. Additional details are provided in the eAppendix and eFigure 1 in Supplement 1.

### Study Population

We included singleton infants born at gestational age 32 weeks or later, encompassing late-preterm and full-term infants. Infants with severe congenital disorders or very low birth weight (<1500 g) were excluded. Infants whose birth weight or family diet type were missing were also excluded. Additional details are provided in the eAppendix in Supplement 1.

### Exposure and Covariates

The primary variable was family dietary pattern, categorized as omnivorous, vegetarian, or vegan, and recorded as a caregiver-reported household-level item at or after the introduction of complementary foods, typically at age 6 months or older. If complementary feeding began earlier, classification was obtained at the next visit at or after age 6 months. Covariates included the child’s sex, gestational age in weeks, parity, mode of delivery (vaginal, cesarean, or instrumental), and maternal age. Geographic-level income and area-level ethnic composition (Arab, Jewish, or multiethnic residents) were derived from the child’s residential address and described in more detail in the eAppendix in Supplement 1. Ethnicity was included to account for potential socioeconomic and cultural differences in dietary practices and health care use that may influence infant growth outcomes. Finally, breastfeeding status (none, partial, or full) was updated at every visit, and cumulative duration was summarized categorically.

### Outcomes

The primary outcome was infant length (in centimeters and World Health Organization [WHO] length-for-age *z* scores [LAZs]). Secondary outcomes included weight and head circumference, measured in kilograms and centimeters, respectively, and WHO age- and sex-specific *z* scores (WHO-ZSs). All WHO-ZSs were calculated using the package Anthro in R, version 4.4.1 (R Foundation for Statistical Computing) for LAZ, weight-for-age *z* score (WAZ), and head circumference *z* score (HCZ). We excluded implausible anthropometric values based on WHO-recommended thresholds^10^ (details provided in the eAppendix in Supplement 1). Children were classified as underweight or overweight if their weight-for-length *z* score (WFLZ) WHO-ZS was less than −2 or greater than 2, respectively. Stunting was defined as an LAZ WHO-ZS less than −2.

Birth weight was a secondary outcome, examined continuously (in kilograms) and categorically as low birth weight (LBW) less than 2.5 kg and high birth weight (HBW) greater than 4.0 kg. Birth weight–for–gestational age centiles (BWGCs) were derived from sex-specific INTERGROWTH-21st Newborn Size at Birth standards^11^ using the R package gigs, classifying infants as small for gestational age (SGA) (<10th BWGC) or large for gestational age (LGA) (>90th BWGC).

### Statistical Analysis

The data analysis was performed between November 17, 2024, and December 6, 2025. Descriptive statistics for participant characteristics and anthropometric outcomes were computed at the first and last visits. Parametric tests were used for group comparisons because of the large sample size, as they are known for their robustness against violations of the normality assumption in large-scale data analyses.^12^ Analyses consisted of pairwise comparisons between dietary groups, including *t* tests for continuous variables and χ^2^ tests for categorical variables. The Bonferroni correction was applied to pairwise comparisons among dietary groups to control for multiple comparisons. Considering the large sample size, statistical significance was defined as a Bonferroni-adjusted *P* < .01.

We used multiple approaches to analyze growth parameters. We calculated monthly means by family diet and sex at routine visit ages (0, 1, 2, 4, 6, 9, 12, 18, and 24 months), corresponding to the vaccination schedule when most measurements occur. We compared first- and last-available measurements for each child. At birth, we recorded initial weight, while initial length and head circumference were obtained within the first 60 days of life. To optimize participant representation, we defined the final visit window for length and weight as occurring between age 700 and 830 days (approximately 24 months), and for head circumference, between age 525 and 650 days (approximately 18 months). These ranges were determined based on visit age distribution within the dataset (eFigure 2 in Supplement 1).

Logistic regression models were used to estimate adjusted odds ratios (AORs) for binary outcomes, including stunting, underweight, and overweight, at birth or early infancy and again at age approximately 24 months. We used linear mixed-effects models to assess the associations between family dietary patterns and repeated measurements of length, weight, and head circumference. To account for potential nonlinear age effects, we specified restricted cubic splines with 5 knots at the 0.050, 0.275, 0.500, 0.725, and 0.950 quantiles a priori.^13^ All models included individual-specific random intercepts. We used 3 sequential models with a progressively expanded adjustment, with covariates selected a priori from established growth determinants.^14^ Model 1 adjusted for age splines and sex; model 2 additionally adjusted for perinatal, maternal, and area-level sociodemographic characteristics; and model 3 further adjusted for birth weight. Generalized additive mixed models were used to estimate age-specific probabilities of stunting, underweight, and overweight over time. These models used the same spline and random-effects structures as the linear models. To assess robustness to follow-up timing, we repeated primary models separately in the sufficient-measurement subset, defined as children with at least 3 length measurements, including at least 1 near age 24 months, and in all other children.

A directed acyclic graph presented in eFigure 3 in Supplement 1 summarizes the hypothesized associations among family diet, birth weight, and child growth. Analyses were conducted using R, version 4.4.1 with the following packages: lme4, mgcv, ggplot2, broom, parameters, and gtsummary.

## Results

### Participant Characteristics

Of the 1 346 089 children in the initial database, 1 198 818 (89.1%) met the inclusion criteria (mean [SD] gestational age, 39.2 [1.5] weeks; 46.8% female and 53.2% male). This group included 1.2% infants in the vegetarian group, 0.3% infants in the vegan group, and 98.5% infants in the omnivorous group. The dataset contained 8 886 904 length, 11 390 292 weight, and 9 334 218 head circumference measurements.

Mothers in the vegan group were older (mean [SD] age at birth, 33.0 [4.9] years) vs those in the vegetarian group (31.9 [5.4]) and omnivorous group (30.0 [5.6] years), had a higher geographic-level income (mean [SD] index, 6.15 [2.33]) vs those in the vegetarian group (5.51 [2.59]) and omnivorous group (4.36 [2.62]), more often resided in predominantly Jewish localities (82%) vs Arab (3%) and multiethnic localities (15%), and had lower parity (mean [SD], 1.4 [0.9]) vs the vegetarian group (1.6 [1.3]) and omnivorous group (1.9 [1.7]) (*P* < .001 for all comparisons) (Table 1). The distribution of infant sex was similar across all groups.

**Table 1.** Maternal Characteristics and Birth Outcomes by Family Dietary Patterns

Characteristic	Infants, No. (%)				*P* value^a^		
	Overall (N = 1 198 818)	Omnivorous group (n = 1 180 690)	Vegetarian group (n = 14 790)	Vegan group (n = 3338)	Vegan vs omnivorous	Vegetarian vs omnivorous	Vegan vs vegetarian
**Maternal characteristics**							
Mother’s age at birth, mean (SD), y	30.0 (5.6)	30.0 (5.6)	31.9 (5.4)	33.0 (4.9)	<.001	<.001	<.001
GLI, mean (SD)^b^	4.38 (2.63)	4.36 (2.62)	5.51 (2.59)	6.15 (2.33)	<.001	<.001	<.001
Area-level ethnic composition^c^							
Arab	197 611 (18.3)	196 149 (18.5)	1370 (10.5)	92 (3.1)	<.001	<.001	<.001
Jewish	666 042 (61.8)	654 124 (61.6)	9495 (72.7)	2423 (81.9)			
Multiethnic	213 912 (19.9)	211 280 (19.9)	2190 (16.8)	442 (14.9)			
Parity, mean (SD)	1.9 (1.7)	1.9 (1.7)	1.6 (1.3)	1.4 (0.9)	<.001	<.001	<.001
Full nursing category							
<1 mo	564 940 (47.1)	558 081 (47.3)	5865 (39.7)	994 (29.8)	<.001	<.001	<.001
1-6 mo	602 148 (50.2)	591 490 (50.1)	8480 (57.3)	2178 (65.3)			
7-11 mo	26 804 (2.2)	26 301 (2.2)	371 (2.5)	132 (4.0)			
≥12 mo	4751 (0.4)	4646 (0.4)	72 (0.5)	33 (1.0)			
Partial nursing category							
<1 mo	202 817 (16.9)	200 658 (17.0)	1867 (12.6)	292 (8.8)	<.001	<.001	<.001
1-6 mo	467 028 (39.0)	461 154 (39.1)	4938 (33.4)	936 (28.0)			
7-11 mo	198 094 (16.5)	194 999 (16.5)	2564 (17.3)	531 (15.9)			
≥12 mo	330 704 (27.6)	323 707 (27.4)	5419 (36.6)	1578 (47.3)			
**Birth outcomes**							
Sex							
Female	560 701 (46.8)	552 037 (46.8)	7015 (47.4)	1649 (49.4)	.03	>.99	.75
Male	638 117 (53.2)	628 653 (53.2)	7775 (52.6)	1689 (50.6)			
Gestational age, mean (SD), wk	39.2 (1.5)	39.2 (1.5)	39.2 (1.5)	39.2 (1.5)	>.99	>.99	>.99
Vaginal birth	934 075 (77.9)	920 523 (78.0)	11 072 (74.9)	2480 (74.3)	<.001	<.001	>.99
Birth weight, mean (SD), kg	3.3 (0.5)	3.3 (0.5)	3.2 (0.5)	3.2 (0.5)	<.001	<.001	<.001
BWGC, mean (SD)	54 (28)	54 (28)	53 (28)	47 (28)	<.001	<.001	<.001
LBW	55 101 (4.6)	54 153 (4.6)	743 (5.0)	205 (6.1)	<.001	.22	.11
SGA	64 814 (6.6)	63 669 (6.6)	860 (7.4)	285 (11.0)	<.001	.03	<.001
HBW	61 625 (5.1)	60 857 (5.2)	654 (4.4)	114 (3.4)	<.001	<.001	.12

Abbreviations: BWGC, birth weight–for–gestation age centile; GLI, geographic-level income; HBW, high birth weight (>4 kg); LBW, low birth weight (<2.5 kg); LGA, large for gestational age (>90 birth weight centile); SGA, small for gestational age (<10 birth weight centile).

^a^ *P* values adjusted (Bonferroni) for multiple comparisons.

^b^ The GLI is an area-based income index (0-10) derived from the child’s residential geographic statistical area; higher values indicate higher neighborhood income.

^c^ Defined by municipality population (Arab, >90% Arab; Jewish, >90% Jewish; multiethnic, >10% to <90%); reflects area, not individual, ethnicity.

Infants in the vegan group had a lower birth weight compared with the omnivorous group (mean [SD], 3.2 [0.5] vs 3.3 [0.5] kg, respectively), with infants in the vegetarian group having intermediate values (mean [SD], 3.2 [0.5] kg). Mean BWGC followed the same pattern, being lowest in the vegan group (mean [SD], 47 [28] vs 53 [28] and 54 [28] in the vegetarian and omnivorous groups, respectively) (*P* < .001 for all comparisons). The incidence of LBW was higher in the vegan group (6.1%) compared with the vegetarian (5.0%) and omnivorous (4.6%) groups (*P* < .001 for vegan vs omnivorous). Conversely, HBW was least common in the vegan group (3.4%) vs the omnivorous group (5.2%) (*P* < .001). Infants who were SGA and LGA exhibited patterns similar to those with LBW and HBW, respectively. Additionally, mothers in the vegan group had significantly longer durations of both full (1-6 months: 65.3% vs 57.3% and 50.1% in the vegetarian and omnivorous groups, respectively) and partial nursing (≥12 months: 47.3% vs 36.6% and 27.4% in the vegetarian and omnivorous groups, respectively) (all *P* < .001).

### Growth Parameters

#### Anthropometric Outcomes at Birth and Early Infancy

Early growth outcomes assessed within the first 60 days of life showed modest variations by family dietary patterns (eTable 1 in Supplement 1), aligning with previously described birth weight patterns. Mean differences in LAZ, WAZ, WFLZ, and HCZ were small (WHO*-*ZS <0.3). Stunting prevalence (omnivorous group, 7.1%; vegetarian group, 7.0%; vegan group, 7.0%) did not differ significantly among dietary groups (Table 2). Infants in the vegan group had a higher odds of being underweight compared with those in the omnivorous group (AOR, 1.37 [95% CI, 1.15-1.63]), while infants in the vegetarian group also showed a slightly increased odds (AOR, 1.21 [95% CI, 1.11-1.32]). Overweight prevalence was low across all groups (omnivorous group, 2.4%; vegetarian group, 2.0%; vegan group, 1.8%), with no significant dietary differences in fully adjusted models (Table 2; eTable 2 in Supplement 1).

**Table 2.** Associations Between Family Dietary Pattern and Nutritional Status Outcomes at First and Last Visits

Outcome and family dietary pattern^a^	Prevalence, No./total No. (%)	Model 1^b^		Model 2^c^	
		AOR (95% CI)	*P* value	AOR (95% CI)	*P* value
**Stunting** ^d^					
First visit					
Omnivorous	72 561/1 026 946 (7.1)	1 [Reference]	NA	1 [Reference]	NA
Vegetarian	926/13 282 (7.0)	1.00 (0.94-1.07)	.95	1.08 (0.98-1.18)	.11
Vegan	207/2966 (7.0)	1.02 (0.89-1.18)	.77	1.11 (0.92-1.34)	.29
Last visit					
Omnivorous	15 527/506 262 (3.1)	1 [Reference]	NA	1 [Reference]	NA
Vegetarian	210/6266 (3.4)	1.10 (0.96-1.26)	.18	1.07 (0.90-1.26)	.45
Vegan	44/1136 (3.9)	1.28 (0.95-1.74)	.10	1.05 (0.71-1.54)	.82
**Underweight** ^d^					
First visit					
Omnivorous	48 240/1 025 497 (4.7)	1 [Reference]	NA	1 [Reference]	NA
Vegetarian	756/13 259 (5.7)	1.19 (1.10-1.38)	<.001	1.21 (1.11-1.32)	<.001
Vegan	214/2962 (7.2)	1.50 (1.30-1.73)	<.001	1.37 (1.15-1.63)	.001
Last visit					
Omnivorous	4083/504 890 (0.8)	1 [Reference]	NA	1 [Reference]	NA
Vegetarian	41 of 6249 (0.7)	0.81 (0.60-1.10)	.18	0.80 (0.55-1.15)	.22
Vegan	12 of 1132 (1.1)	1.33 (0.75-2.36)	.33	1.06 (0.50-2.23)	.89
**Overweight** ^d^					
First visit					
Omnivorous	24 191/1 025 497 (2.4)	1 [Reference]	NA	1 [Reference]	NA
Vegetarian	262/13 259 (2.0)	0.86 (0.76-0.97)	.02	0.93 (0.80-1.07)	.32
Vegan	53/2962 (1.8)	0.80 (0.61-1.05)	.10	0.86 (0.61-1.2)	.37
Last visit					
Omnivorous	19 607/504 890 (3.9)	1 [Reference]	NA	1 [Reference]	NA
Vegetarian	231/6249 (3.7)	0.95 (0.83-1.09)	.46	1.01 (0.86-1.18)	.89
Vegan	39/1132 (3.4)	0.88 (0.64-1.22)	.45	0.91 (0.61-1.35)	.63

Abbreviations: AOR, adjusted odds ratio; NA, not applicable.

^a^ First visit is defined as birth (age 0), while initial measurements for length were obtained within the first 60 days of life. The last visit for length and weight was between age 700 and 810 days (approximately 24 months).

^b^ Model 1 was adjusted for sex and age.

^c^ Model 2 was additionally adjusted for maternal age, gestational week, birth type, parity, geographic-level income measure, and area-level ethnic composition. For last visit outcomes, model 2 was also adjusted for breastfeeding status. Full model specifications are presented in eTable 2 in Supplement 1.

^d^ Outcome definitions (based on World Health Organization standards): stunting, length-for-age *z* score less than −2; underweight, weight-for-length *z* score less than −2; overweight, weight-for-length *z* score greater than 2.

#### Growth Outcomes at Follow-Up

At approximately age 24 months, growth differences among dietary groups were minimal (WHO-ZS ≤0.2). Stunting prevalence declined across dietary groups (omnivorous group, 3.1%; vegetarian group, 3.4%; vegan group, 3.9%) and did not differ significantly. Underweight and overweight prevalence was low and nonsignificant across groups. Adjusted ORs did not differ among groups for any outcome (Table 2; eTable 2 in Supplement 1).

#### Longitudinal Patterns

**Figure 1.**
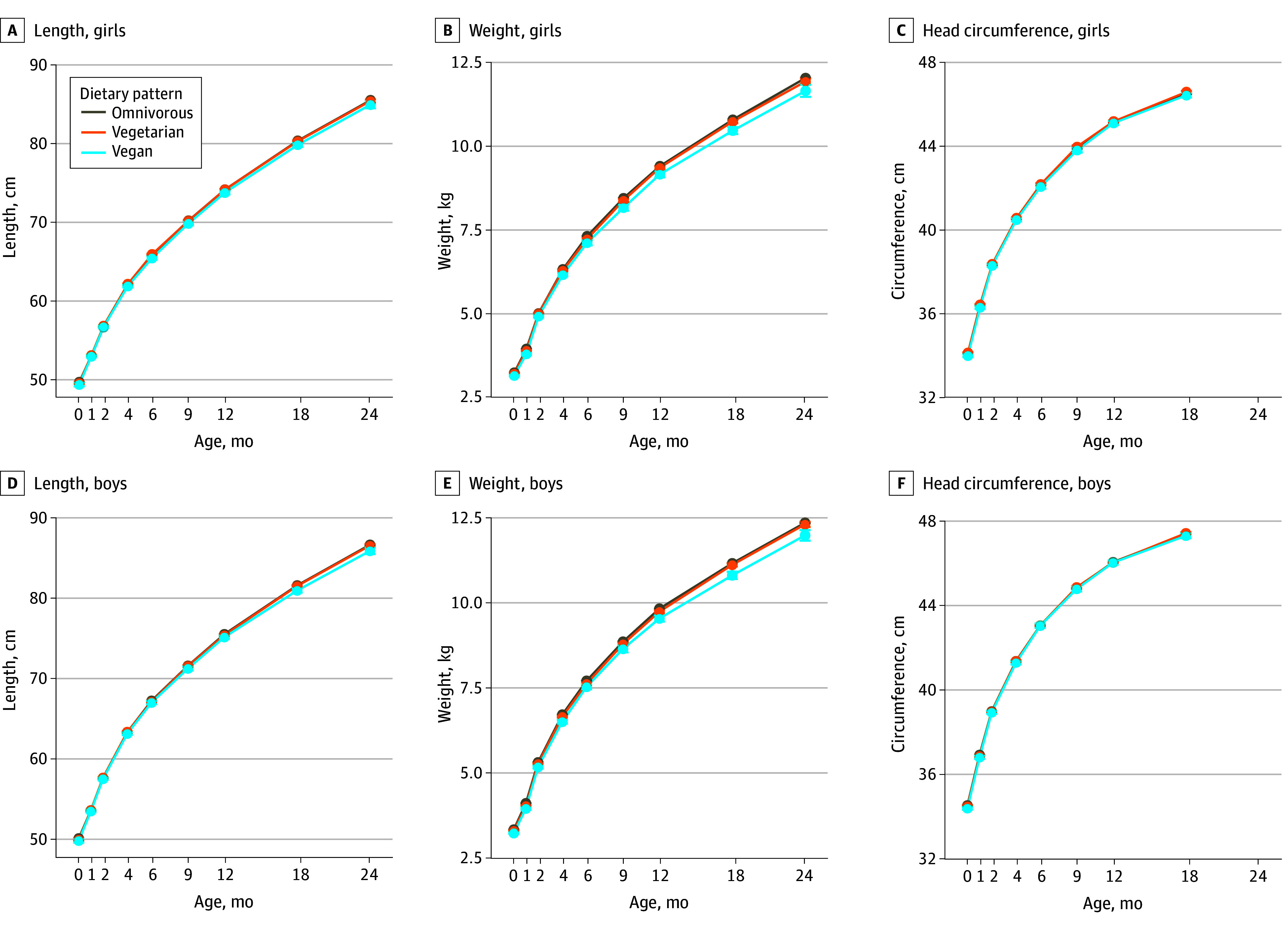
Crude Monthly Means of Anthropometry at Routine Ages by Family Dietary Patterns Points and error bars indicate bin means with 95% CIs.

Monthly crude means showed small between-group differences in length and weight, mirrored in WHO-ZSs, with no differences in head circumference (Figure 1; eFigure 4 in Supplement 1; eTable 3 in Supplement 2). Continuous anthropometric trajectories over the first 24 months of life, adjusted for maternal and birth-related factors, revealed modest, but persistent differences among dietary groups. In minimally adjusted models, children in the vegan group had lower LAZ (β = −0.06 [95% CI, −0.10 to −0.03]), WAZ (β = −0.19 [95% CI, −0.22 to −0.16]), HCZ (β = −0.04 [95% CI, −0.07 to −0.01]), and WFLZ (β = −0.21 [95% CI, −0.24 to −0.18]) compared with the omnivorous group (Table 3). These differences were attenuated slightly, with length crossing 0, after additional adjustment for birth weight (model 3, vegan vs omnivorous group: LAZ, β = 0.06 [95% CI, 0.02-0.09]; WAZ, β = −0.01 [95% CI, −0.04 to 0.01]; WFLZ, β = −0.07 [95% CI, −0.10 to −0.04]). Children in the vegetarian group showed similar patterns, with outcomes generally falling between the vegan and omnivorous groups.

**Table 3.** Associations Between Family Dietary Patterns and Infant Growth Indicators in Adjusted Mixed-Effects Models^a^

Characteristic and family dietary pattern	Model 1^b^		Model 2^c^		Model 3^d^	
	β (95% CI)	*P* value	β (95% CI)	*P* value	β (95% CI)	*P* value
Length						
Omnivorous	1 [Reference]	NA	1 [Reference]	NA	1 [Reference]	NA
Vegan	−0.16 (−0.24 to −0.09)	<.001	−0.18 (−0.26 to −0.09)	<.001	0.13 (0.05 to 0.20)	<.001
Vegetarian	0.05 (0.01 to 0.09)	.006	0.00 (−0.04 to 0.04)	.89	0.10 (0.06 to 0.13)	<.001
LAZ						
Omnivorous	1 [Reference]	NA	1 [Reference]	NA	1 [Reference]	NA
Vegan	−0.06 (−0.10 to −0.03)	<.001	−0.08 (−0.12 to −0.04)	<.001	0.06 (0.02 to 0.09)	<.001
Vegetarian	0.02 (0.01 to 0.04)	.002	0.00 (−0.02 to 0.02)	.86	0.04 (0.03 to 0.06)	<.001
Weight						
Omnivorous	1 [Reference]	NA	1 [Reference]	NA	1 [Reference]	NA
Vegan	−0.15 (−0.18 to −0.13)	<.001	−0.12 (−0.15 to −0.09)	<.001	0.00 (−0.03 to 0.02)	.75
Vegetarian	−0.04 (−0.05 to −0.03)	<.001	−0.03 (−0.04 to −0.01)	<.001	0.01 (0.00 to 0.02)	.06
WAZ						
Omnivorous	1 [Reference]	NA	1 [Reference]	NA	1 [Reference]	NA
Vegan	−0.19 (−0.22 to −0.16)	<.001	−0.17 (−0.20 to −0.14)	<.001	−0.01 (−0.04 to 0.01)	.27
Vegetarian	−0.05 (−0.06 to −0.04)	<.001	−0.04 (−0.06 to −0.03)	<.001	0.01 (0.00 to 0.02)	.19
WFLZ						
Omnivorous	1 [Reference]	NA	1 [Reference]	NA	1 [Reference]	NA
Vegan	−0.21 (−0.24 to −0.18)	<.001	−0.13 (−0.16 to −0.10)	<.001	−0.07 (−0.10 to −0.04)	<.001
Vegetarian	−0.08 (−0.10 to −0.07)	<.001	−0.05 (−0.06 to −0.03)	<.001	−0.02 (−0.04 to −0.01)	.001
Head circumference						
Omnivorous	1 [Reference]	NA	1 [Reference]	NA	1 [Reference]	NA
Vegan	−0.04 (−0.08 to 0.00)	.03	−0.08 (−0.12 to −0.03)	<.001	0.07 (0.03 to 0.11)	<.001
Vegetarian	0.03 (0.01 to 0.05)	.002	0.01 (−0.01 to 0.03)	.34	0.06 (0.04 to 0.08)	<.001
HCZ						
Omnivorous	1 [Reference]	NA	1 [Reference]	NA	1 [Reference]	NA
Vegan	−0.04 (−0.07 to −0.01)	.02	−0.06 (−0.10 to −0.03)	<.001	0.05 (0.02 to 0.08)	<.001
Vegetarian	0.02 (0.01 to 0.03)	.007	0.01 (−0.01 to 0.02)	.45	0.05 (0.03 to 0.06)	<.001

Abbreviations: HCZ, head circumference *z* score; LAZ, length-for-age *z* score; NA, not applicable; WAZ, weight-for-age *z* score; WFLZ, weight-for-length *z* score.

^a^ The mixed-effects models used 5 knots for age (0.050, 0.275, 0.500, 0.725, and 0.950 quantiles).

^b^ Model 1 was adjusted for the child’s age and sex.

^c^ Model 2 included additional adjustments for maternal age, gestational week, birth type, parity, breastfeeding status, geographic-level income measure, and area-level ethnic composition.

^d^ Model 3 incorporated further adjustments for birth weight.

**Figure 2.**
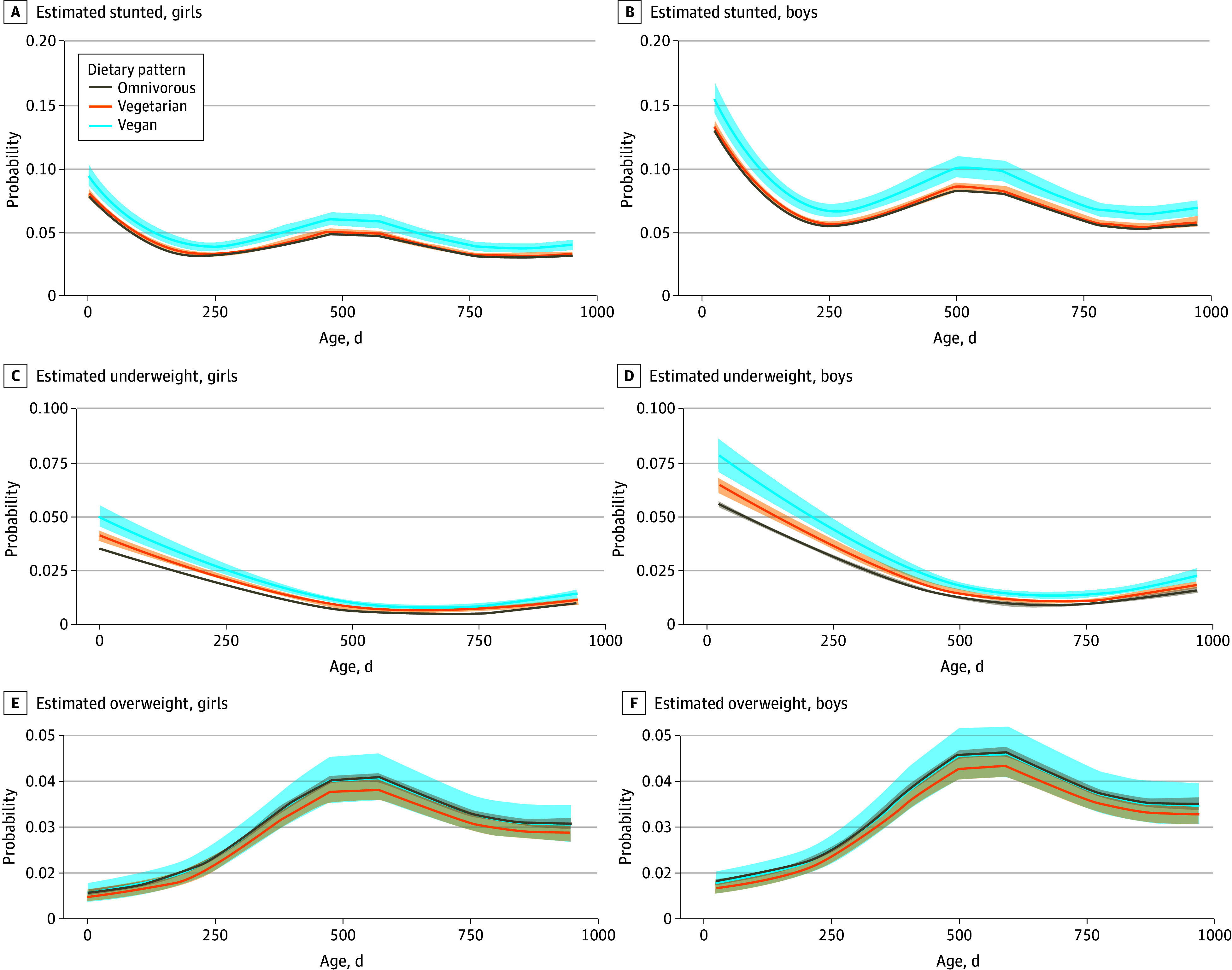
Estimated Probabilities of Stunting, Underweight, and Overweight Across the First 24 Months of Life by Family Dietary Patterns Solid lines indicate population-average probabilities estimated by logistic generalized additive mixed models (with child-specific random intercepts excluded), adjusted for maternal age, gestational week, birth type, parity, breastfeeding duration, geographic-level income, and area-level ethnic composition. Shading indicates Wald 99% CIs.

Generalized additive mixed model–estimated probabilities showed that dietary group differences decreased over time (Figure 2). Stunting probability was slightly higher in infants in the vegan group but remained modest and stable overall. Although underweight-estimated probability was initially higher in the vegan group (Table 2), this probability significantly decreased, closely aligning with omnivorous group levels by age 24 months. The probability of being overweight was consistently lowest in the vegetarian group throughout the follow-up period.

In robustness tests, associations between family dietary patterns and outcomes remained consistent across sufficient-measurement subgroups. Small differences in length and LAZ among dietary groups and similar AORs for stunting, underweight, and overweight were observed (eTables 4-6 in Supplement 1).

## Discussion

This nationwide, longitudinal cohort study in more than 1 million children found that growth trajectories across family vegan, vegetarian, and omnivorous dietary patterns were similar. On average, infants in vegan households were less than 0.5 cm shorter and approximately 100 g lighter at birth compared with their counterparts from omnivorous households. These differences translated into WHO-ZSs of less than 0.2 and were not clinically meaningful. Throughout the first 24 months of life, mean length and weight of infants from vegan and vegetarian households tracked almost indistinguishably from those of omnivorous households, with group contrasts consistently showing WHO-ZSs of 0.2 or less; these modest gaps virtually disappeared after adjustment for birth weight. The primary divergence emerged at distributional extremes, with infants in vegan households experiencing a higher odds of stunting and underweight, especially in early infancy.

However, these outcomes were rare at age 24 months, and diet-associated AOR differences narrowed with age, becoming minimal by that time. Overall, family dietary patterns showed minimal association with mean growth, though the small excess of extreme outcomes in the vegan group may warrant continued monitoring.

Historically, research on maternal plant-based diets and birth outcomes has focused mainly on vegetarian rather than vegan diets and was often conducted in Asian populations, limiting generalizability.^15^ A 2024 meta-analysis found that infants of mothers with a vegan dietary pattern weighed approximately 240 g less at birth and had a higher odds of LBW (OR, 2.71 [95% CI, 1.24-5.95]).^16^

Studies from Western countries show mixed results with regard to growth and nutritional adequacy among young children consuming plant-based diets. Some studies reported comparable growth metrics among children of vegan, vegetarian, and omnivore households,^17,18^ while others noted lower LAZ, body mass index WHO-ZS, other anthropometrics, and bone mineral content among children from vegan households.^19^ Consistent with these mixed findings, we found that infants and toddlers from vegan households had a mean growth similar to those from omnivorous households, with slightly higher, but uncommon and age-declining odds of underweight. Given that overweight and obesity are more common concerns in developed countries,^20^ the similar growth patterns and possible protection against excessive weight gain seen with plant-based diets may offer useful insights for health and nutrition guidance.

Birth weight at delivery may partially explain the observed growth differences. Adjusting for birth weight attenuated observed disparities, suggesting that part of the association may reflect smaller size at birth rather than differential postnatal growth alone. This finding aligns with earlier Israeli studies that reported lower birth weight and birth weight centiles and increased prevalence of SGA among mothers following a vegan diet.^21,22^ The elevated SGA AOR in mothers following a vegan diet may be partially attributable to lower prepregnancy body mass index, a recognized SGA risk factor.^23^ Another possible explanation is reduced protein intake commonly observed among individuals following a vegan diet,^24^ although clinical implications in otherwise well-nourished groups remain uncertain.^25^ As we lacked data on maternal nutrition and detailed maternal dietary intake, these mechanistic explanations should be viewed with caution.

Breastfeeding duration was longer in the vegan and vegetarian groups vs the omnivorous group, consistent with a previous report.^26^ Although prolonged breastfeeding slightly increased stunting AOR, it was negatively associated with overweight at age 24 months (eTable 2 in Supplement 1), aligning with results of other studies.^27^ Given breastfeeding’s health benefits, including improved cardiometabolic outcomes, reduced infectious morbidity,^28^ enhanced child development,^29^ and reduced all-cause mortality,^28,30^ breastfeeding seems advantageous overall, despite a modest associated increase in stunting AOR.

Recent literature has indicated that well-planned vegan diets may meet children’s nutritional needs and support healthy growth with appropriate supplementation (eg, vitamin B_12_) and nutrient monitoring.^8^ Children following a vegan diet typically exhibit lower cholesterol levels and a reduced prevalence of overweight and obesity compared with children following an omnivorous diet.^31^ Nonetheless, poorly planned vegan diets can result in deficiencies, particularly in vitamin B_12_, vitamin D, calcium, iron, iodine, and omega-3 fatty acids.^8^ Furthermore, modern vegan diets in Western countries often include highly processed alternatives to animal-based foods, which are not recommended and may replace minimally processed plant foods.^32^ This evidence highlights the importance of nutritional counseling and pediatric follow-up of infants and children from vegan households, as well as pregnant and breastfeeding mothers. Vegan diets are generally higher in fiber, are lower in saturated fats, and have favorable nutrient profiles, possibly explaining their association with lower obesity odds.^33^ However, since nutritional adequacy varies widely among families, future research should assess actual intake, supplementation, and biomarkers to better characterize diet quality in children from vegan households.

### Strengths and Limitations

This study’s strengths included its large, ethnically diverse cohort of more than 1 million children, including thousands from vegetarian and vegan households, making it one of the largest studies on this topic. The population broadly represented Israeli infants with minimal selection bias, including all demographic population groups, since FCCs are nationwide and free for all Israeli citizens and residents. Notably, 70% of Israeli infants attend MOH FCCs and are included in this database.^34^ The large sample provided statistical power to detect rare outcomes such as underweight and stunting, which smaller studies often could not assess reliably. Anthropometry data were measured by trained FCC nurses following standardized MOH protocols. Prospective, repeatedly measured parameters stored in an electronic registry enabled assessment of growth trajectories over time while reducing recall bias.

While this study has provided valuable insights, there are several limitations. First, coverage was limited to MOH- and municipality-operated FCCs (approximately 70% of infants). Although FCC attendance in Israel exceeds 95%, we lacked data to compare infants seen at health maintenance organization–operated centers; therefore, differences between included and nonincluded infants and potential selection bias cannot be ruled out. Second, most visits occurred in the first year of life, and 37% of children lacked measurements at age 24 months or older. However, differences in confounders between children with and without later measurements were small, and robustness checks produced similar estimates (eTables 4-6 in Supplement 1). Third, family dietary pattern was a single caregiver-reported household measure, not repeated longitudinally, and did not capture individual maternal or infant intake. Data on complementary feeding; detailed infant diet; and detailed maternal diet, including postweaning intake and supplement use, were unavailable, limiting assessment of nutritional exposures. However, caregiver-reported household dietary patterns may have reflected caregiver-infant dietary concordance during the first 24 months.^35,36^ Fourth, the definitions of vegetarian and vegan were not standardized, and vegetarian subtypes (eg, ovo-lacto vegetarianism, pescatarianism) were not distinguished, yielding a clearly defined vegan group and a more heterogeneous vegetarian group, as expected. Fifth, due to the lack of a standardized definition, partial breastfeeding duration categories were heterogeneous and may have been subject to misclassification. Sixth, geographic-level income and residential ethnic composition were derived from area-level data, which may not fully represent individual socioeconomic or ethnic groups. Seventh, detailed information on maternal diet, prepregnancy body mass index, height, and paternal factors, all known predictors of growth, was also unavailable. Eighth, follow-up extended only until age 30 months, so longer-term growth trends were not evaluated. Finally, the dataset lacked information on maternal smoking before and during pregnancy. Smoking is a well-established risk factor for LBW^37^; however, the smoking prevalence during pregnancy in Israel is low,^38^ making it reasonable to assume that potential confounding from smoking was minimal.

## Conclusions

This cohort study found that family vegan, vegetarian, and omnivorous dietary patterns were associated with minimal growth differences among infants, and a vegan or vegetarian diet was associated with a higher odds of underweight, particularly in early infancy. However, these outcomes remained rare across groups and became less pronounced by age 24 months. In developed countries, the growth trajectories observed in children from vegan households seem broadly reassuring. These findings suggest that a vegan family dietary pattern may support healthy growth in infancy when appropriately planned. Nutritional counseling during pregnancy and early infancy, combined with regular growth monitoring, may help ensure optimal infant development.

## Notes

Supplement 1. **eAppendix.** Data Sources and Data Management Protocol **eFigure 1.** Flowchart of Cohort Selection and Exclusions **eFigure 2.** Distribution of Visit Ages for Weight, Length, and Head Circumference Measurements During the First 2 Years of Life **eFigure 3.** Illustrative Directed Acyclic Graph **eFigure 4.** Crude Monthly Means of WHO *z* Scores by Family Dietary Patterns **eTable 1.** Mean Change in Infant Anthropometry Between First and Last Visit, by Family Dietary Patterns **eTable 2.** Full Model Coefficients for Stunting, Underweight, and Overweight at First and Last Visits **eTable 4.** Sensitivity Analysis of Associations Between Family Dietary Pattern and Infant Length and Length-for-Age *z* Scores in Subgroups Defined by Measurement Sufficiency **eTable 5.** Sensitivity Analysis of Associations Between Family Dietary Patterns and Growth Outcomes in Subgroups Defined by Measurement Sufficiency **eTable 6.** Baseline Characteristics by Follow-Up Completeness: Sufficient vs Limited Lenth Measurements

Supplement 2. **eTable 3.** Mean Anthropometric Measures and WHO *z* Scores From Birth to Age 18 Months Among Infants, by Sex and Family Dietary Pattern

Supplement 3.
Data Sharing Statement
