# Error in Key Points

**DOI:** 10.1001/jamanetworkopen.2026.4763

**Published:** 2026-03-09

In the Original Article titled “Growth Trajectories in Infants From Families With Plant-Based or Omnivorous Dietary Patterns,”^1^ published February 5, 2026, the Key Points Findings should not have stated that there were increased odds of stunting among children in vegan households. This article has been corrected.^1^
