# Supplementary material for: Growth Trajectories in Infants From Families With Plant-Based or Omnivorous Dietary Patterns
Source: JAMA Netw Open. 2026 Feb 5;9(2):e2557798. doi: 10.1001/jamanetworkopen.2025.57798 (PMC12878416; doi:10.1001/jamanetworkopen.2025.57798)
Supplement: Supplement 1. — eAppendix. Data Sources and Data Management Protocol eFigure 1. Flowchart of Cohort Selection and Exclusions eFigure 2. Distribution of Visit Ages for Weight, Length, and Head Circumference Measurements During the First 2 Years of Life eFigure 3. Illustrative Directed Acyclic Graph eFigure 4. Crude Monthly Means of WHO z Scores by Family Dietary Patterns eTable 1. Mean Change in Infant Anthropometry Between First and Last Visit, by Family Dietary Patterns eTable 2. Full Model Coefficients for Stunting, Underweight, and Overweight at First and Last Visits eTable 4. Sensitivity Analysis of Associations Between Family Dietary Pattern and Infant Length and Length-for-Age z Scores in Subgroups Defined by Measurement Sufficiency eTable 5. Sensitivity Analysis of Associations Between Family Dietary Patterns and Growth Outcomes in Subgroups Defined by Measurement Sufficiency eTable 6. Baseline Characteristics by Follow-Up Completeness: Sufficient vs Limited Lenth Measurements [file jamanetwopen-e2557798-s001.pdf]

Supplemental Online Content

Avital K, Fliss-Isakov N, Shahar DR, et al. Growth trajectories in infants from families with plant-based or omnivorous dietary patterns. *JAMA Netw Open*. 2026;9(2):e2557798.
doi:10.1001/jamanetworkopen.2025.57798

eAppendix. Data Sources and Data Management Protocol

eFigure 1. Flowchart of Cohort Selection and Exclusions

eFigure 2. Distribution of Visit Ages for Weight, Length, and Head Circumference Measurements During the First 2 Years of Life

eFigure 3. Illustrative Directed Acyclic Graph

eFigure 4. Crude Monthly Means of WHO *z* Scores by Family Dietary Patterns

eTable 1. Mean Change in Infant Anthropometry Between First and Last Visit, by Family Dietary Patterns

eTable 2. Full Model Coefficients for Stunting, Underweight, and Overweight at First and Last Visits

eTable 4. Sensitivity Analysis of Associations Between Family Dietary Pattern and Infant Length and Length-for-Age *z* Scores in Subgroups Defined by Measurement Sufficiency

eTable 5. Sensitivity Analysis of Associations Between Family Dietary Patterns and Growth Outcomes in Subgroups Defined by Measurement Sufficiency

eTable 6. Baseline Characteristics by Follow-Up Completeness: Sufficient vs Limited Length Measurements

This supplemental material has been provided by the authors to give readers additional information about their work.

© 2026 Avital K et al. *JAMA Network Open*.

eAppendix. Data Sources and Data Management Protocol

Data Sources and Measures

1. Family Care Center records:
The Family Care Center records (FCC) database includes electronic records from all well-baby clinics in Israel. These clinics provide routine pediatric follow-up for children from birth to age 6, including scheduled visits at birth and at 1, 2, 4, 6, 9, 12, 18, 24, 36, 48, and 60 months of age. At each visit, trained nurses document:

- Anthropometric measurements (weight, length/height, and head circumference)
- Developmental milestones (not included in the analytic dataset)
- Breastfeeding status (none, partial, or exclusive).

On the 6-month visit, family dietary patterns are classified by the clinic nurse as omnivore, vegetarian, or vegan based on parental reports. The clinic form did not provide definitions of "vegetarian" or "vegan"; responses reflect caregiver-reported household classification and did not ascertain maternal diet during pregnancy or lactation, or the infant's detailed post-weaning intake.

The FCC database included a total of 12,124,758 growth-monitoring visits. Visits occurring after 2.5 years of age (defined as >913 days) were excluded because the final routine vaccination is typically administered at 2 years. This restriction was applied to reduce selection bias related to irregular follow-up in older children and to better represent the general infant population, resulting in 11,507,015 visits retained for analysis.

2. National Birth Registry:
The National Birth Registry (NBR) includes all live births in Israel and provides perinatal and demographic information, including:

- Maternal age
- Gestational age
- Birth weight
- Birth type: vaginal, cesarean or instrumental b,
- Parity
- Infant sex
- Geographic-level data, including:
    - Geographic-level income measure (GLI), determined according to the geographic statistical area of the child's residence using data from the Israel Central Bureau of Statistics.
    - Area-level ethnic composition, calculated by linking municipality of residence to census-based population data to derive the proportion of Arab and Jewish residents. Municipalities were categorized as Jewish (>90%

© 2026 Avital K et al. *JAMA Network Open*.

Jewish), mixed (>10% to <90% Jewish), or Arab (>90% Arab).

Records from the FCC and NBR were linked using a unique identifier.

Anthropometric Data Cleaning

Z-scores were calculated for all anthropometric measures using the *anthro* package in R, based on WHO 2006/2007 growth standards. Implausible values were removed using the following WHO-recommended cutoffs:

- Weight-for-age: z < −6.0 or > +5.0
- Length/height-for-age: z < −6.0 or > +6.0
- Weight-for-length (WFL), BMI-for-age, and head circumference: z < −5.0 or > +5.0

Validation and Imputation of Birth Weight

Birth weight was recorded in both the FCC and birth registry databases. A multi-step protocol was used to harmonize the two values:

1. If the birth registry value was missing, the value from the FCC database at the first visit (age 0 days) was used.
2. If both sources were available:
    - When the absolute difference was <100 g, the registry value was retained.
    - When the difference was ≥100 g, both values were evaluated for plausibility:
        - Implausible values were defined using gestational-age–specific reference curves (Intergrowth-21st standards).
        - The value deemed more plausible based on gestational age and consistency with subsequent growth records was retained.

Exclusion Process

Infants were excluded if they met any of the following conditions:

- Multiple gestation (n = 55,144)
- Gestational age <31 weeks (n = 5,174)
- Missing family dietary pattern (n = 74,277)
- Missing infant sex (n = 8,436)
- Very low birth weight (<1.5 kg) (n = 1,955)

These exclusions were applied sequentially to the linked dataset.

Variable Cleaning

The following values were recoded as missing (NA) if they fell outside of acceptable

© 2026 Avital K et al. *JAMA Network Open*.

ranges:

Variable	Criteria for recoding to NA
**Maternal age**	<12 y or >55 y
**Gestational age**	<23 or >43 weeks
**Income decile**	Values outside 1–10
**Parity**	>15
**Mode of delivery**	Missing or unclassifiable
**Infant sex**	Missing or ambiguous

Final Analytic Sample

After all linkage, cleaning, and exclusions, the final analytic sample included 1,198,818 infants. Based on dietary classification, these were distributed as follows:

- Omnivore families: 1,180,690
- Vegetarian families: 14,790
- Vegan families: 3,338

These infants contributed a total of 11,507,015 valid visits used in longitudinal growth analyses.

© 2026 Avital K et al. *JAMA Network Open*.

eFigure 1. Flowchart of Cohort Selection and Exclusions

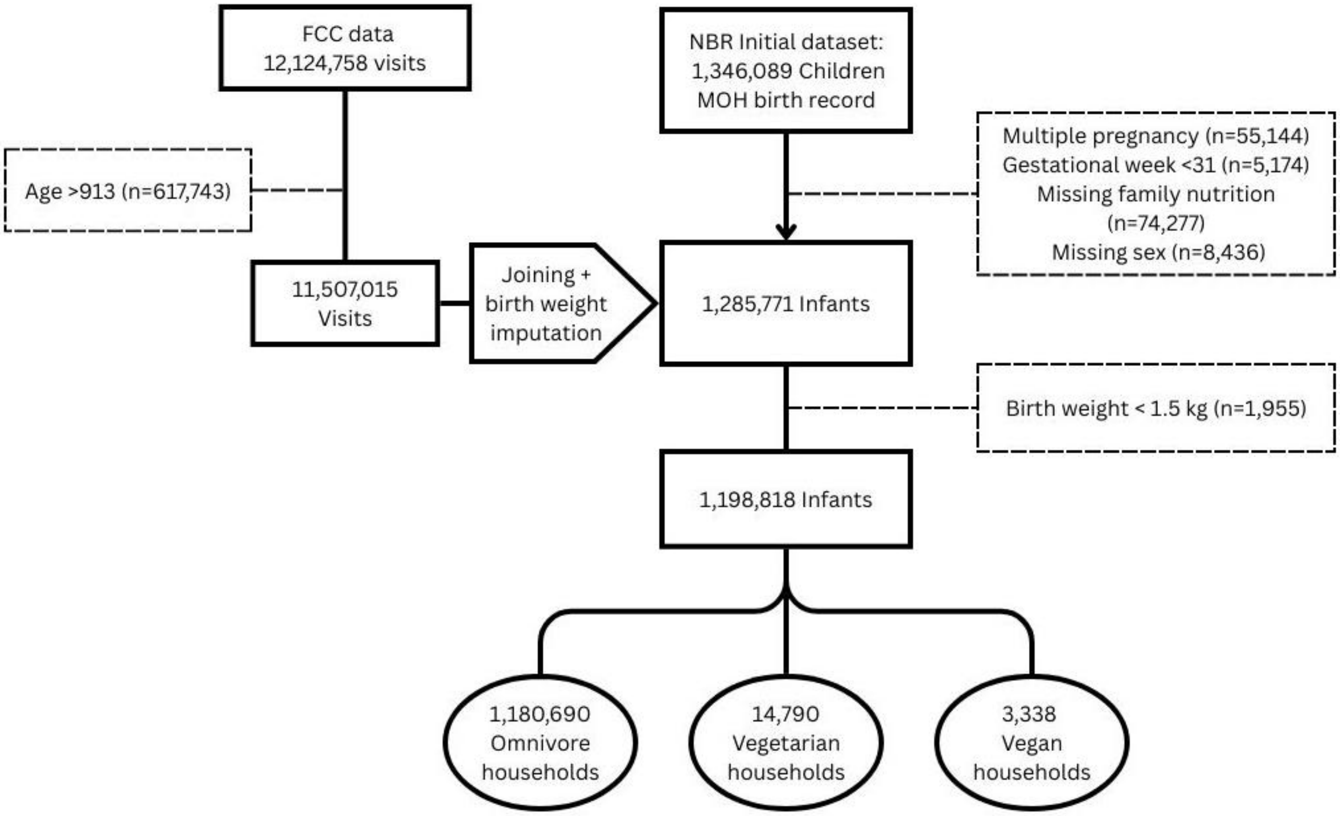

FCC: Family Care Center records database; NBR: National Birth Registry; See eAppendix 1 for more information on the databases

© 2026 Avital K et al. *JAMA Network Open*.

eFigure 2. Distribution of Visit Ages for Weight, Length, and Head Circumference Measurements During the First 2 Years of Life

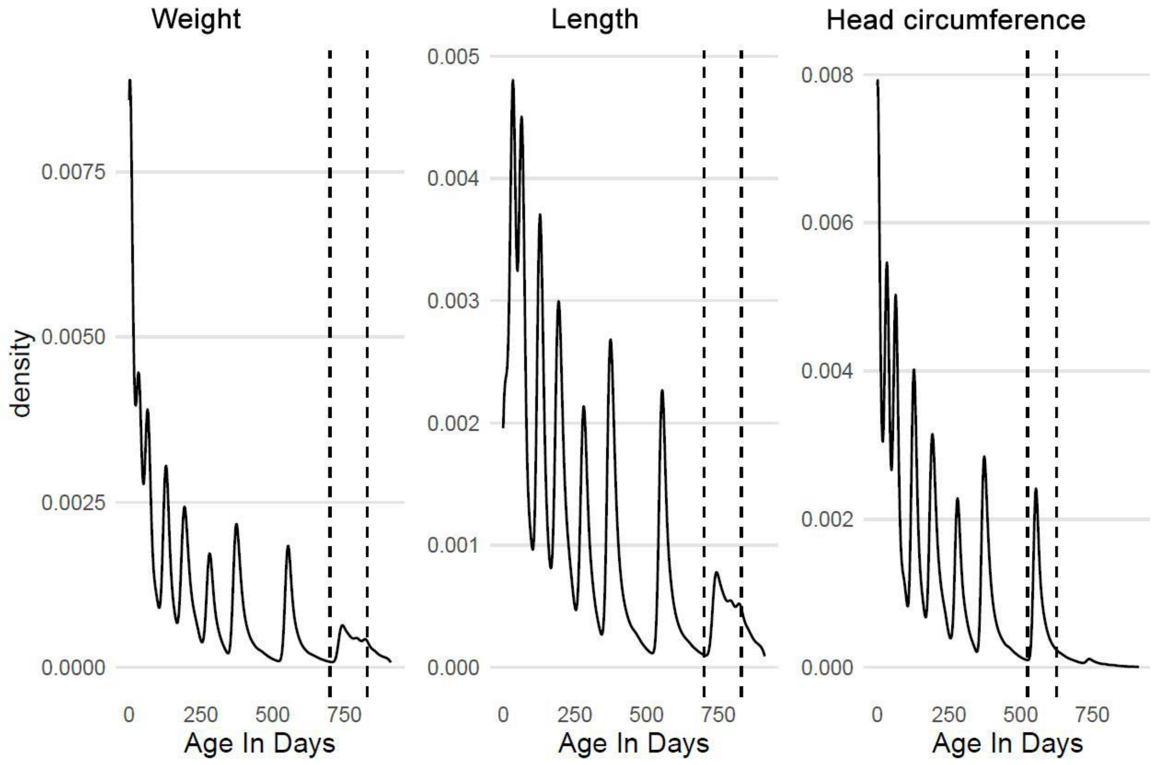

Kernel density plots illustrate the timing of visits contributing anthropometric data. Vertical dashed lines mark the age ranges used to define the final measurement window for each parameter: 700–830 days for weight and length, and 525–650 days for head circumference. These windows were selected to optimize sample representation near age 2 years (or 1.5 years for head circumference).

© 2026 Avital K et al. *JAMA Network Open*.

eFigure 3. Illustrative Directed Acyclic Graph

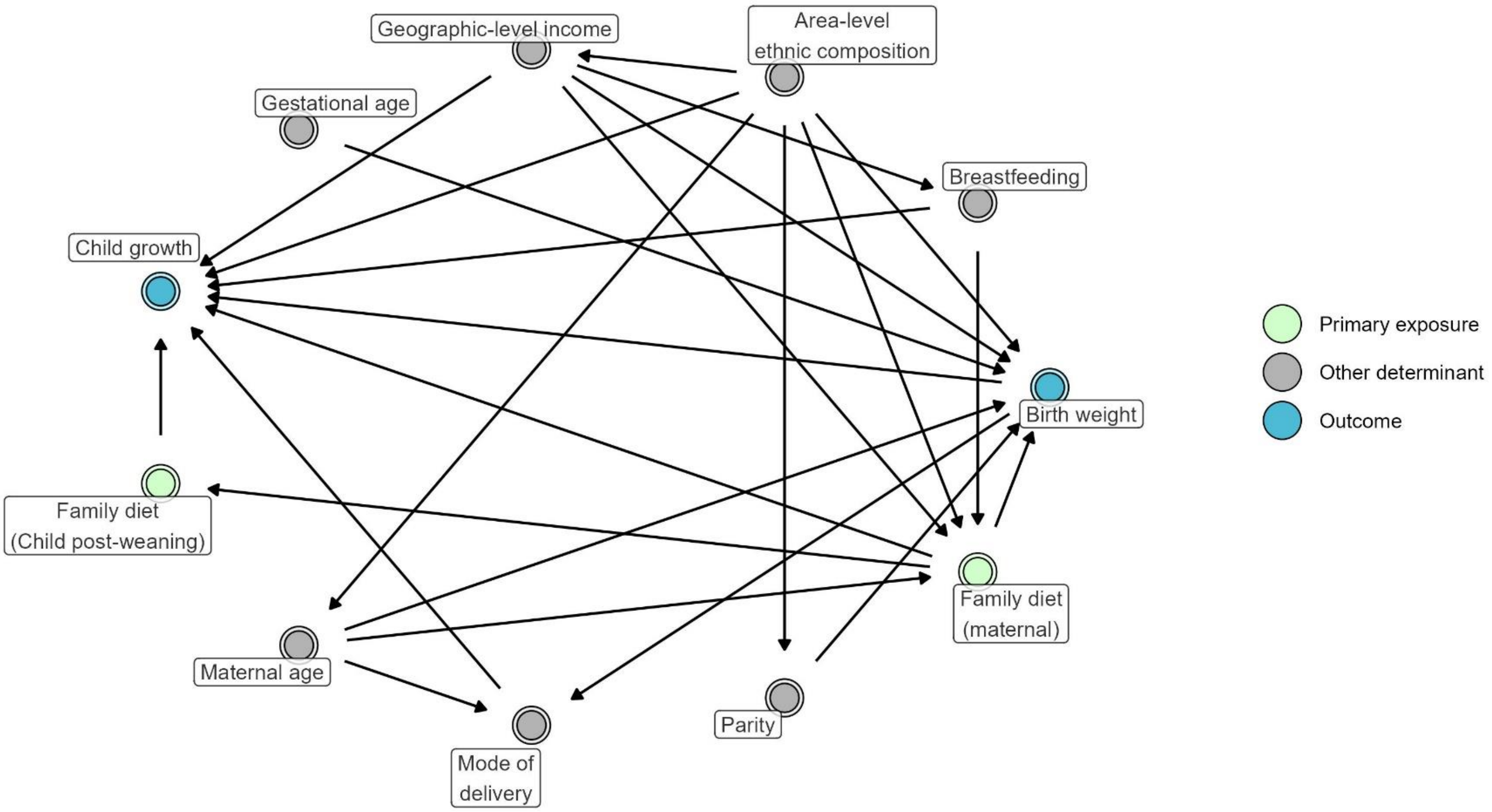

© 2026 Avital K et al. *JAMA Network Open*.

eFigure 4. Crude Monthly Means of WHO *z* Scores by Family Dietary Patterns

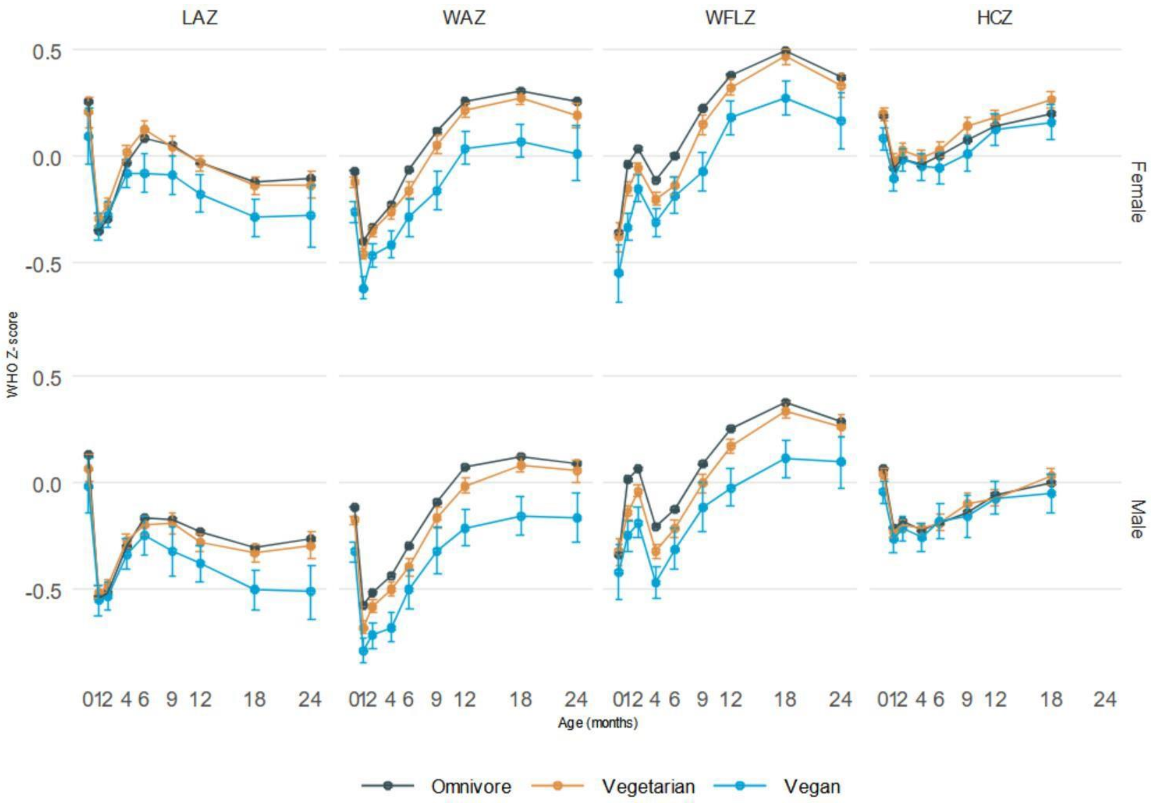

Monthly means WHO z-scores for length/height, weight, head circumference, and weight-for-length at routine ages, with 95% CIs for the mean; colors denote family diet groups (omnivore, vegetarian, vegan).

Abbreviations: WAZ, weight-for-age z score; LAZ, length-for-age z score; WFLZ, weight-for-length z score; HCZ, head circumference z score.

© 2026 Avital K et al. *JAMA Network Open*.

eTable 1. Mean Change in Infant Anthropometry Between First and Last Visit, by Family Dietary Patterns [a]

Parameter	Measurement[b]	Overall	Omnivore	Vegetarian	Vegan	P (V-O)[c]	P (Veg-O)[c]	P (V-Veg)[c]
**Weight (kg)**	First visit	3.27 (0.46)	3.27 (0.46)	3.24 (0.45)	3.17 (0.45)	<0.001	<0.001	<0.001
	Last visit	12.49 (1.46)	12.50 (1.46)	12.45 (1.43)	12.23 (1.45)	<0.001	0.074	<0.001
**WAZ**	First visit	-0.10 (0.98)	-0.09 (0.98)	-0.15 (0.98)	-0.29 (0.98)	<0.001	<0.001	<0.001
	Last visit	0.19 (0.95)	0.19 (0.95)	0.16 (0.94)	0.02 (0.96)	<0.001	0.038	<0.001
**Height (cm)**	First visit	52.29 (2.98)	52.30 (2.98)	52.17 (2.98)	51.93 (3.00)	<0.001	<0.001	<0.001
	Last visit	87.4 (3.4)	87.4 (3.4)	87.2 (3.3)	86.7 (3.3)	<0.001	0.004	<0.001
**LAZ**	First visit	-0.32 (1.16)	-0.32 (1.16)	-0.29 (1.16)	-0.33 (1.13)	>0.9	0.043	0.5
	Last visit	-0.11 (1.02)	-0.11 (1.02)	-0.16 (1.00)	-0.31 (1.00)	<0.001	<0.001	<0.001
**WFLZ**	First visit	-0.12 (1.11)	-0.12 (1.11)	-0.24 (1.11)	-0.39 (1.13)	<0.001	<0.001	<0.001
	Last visit	0.32 (0.96)	0.32 (0.96)	0.31 (0.95)	0.22 (0.97)	0.014	>0.9	0.11
**Head circ. (cm)**	First visit	34.42 (1.41)	34.42 (1.41)	34.43 (1.42)	34.33 (1.45)	0.008	>0.9	0.010
	Last visit	47.09 (1.36)	47.09 (1.36)	47.13 (1.36)	47.06 (1.33)	>0.9	0.2	>0.9
**HCZ**	First visit	0.11 (1.07)	0.11 (1.07)	0.10 (1.07)	0.00 (1.06)	<0.001	>0.9	<0.001
	Last visit	0.10 (0.96)	0.10 (0.96)	0.13 (0.97)	0.10 (0.93)	>0.9	0.061	>0.9

[a]All values are presented as mean (SD);

[b]First visit defined as birth (age = 0), while initial measurements for length and head circumference were obtained within the first 60 days of life. The last visit for length and weight was between 700 and 810 days of age (~2 years), and for head circumference, between 525 and 650 days (~1.5 years).

[c]P values adjusted (Bonferroni) for multiple comparisons: P (V-O): Vegan vs Omnivore, P (Veg-O): Vegetarian vs Omnivore, P (V-Veg): Vegan vs Vegetarian.

Abbreviations: WAZ, weight-for-age z score; LAZ, length-for-age z score; WFLZ, weight-for-length z score; Head circ, head circumference; HCZ, head circumference z score.

© 2026 Avital K et al. *JAMA Network Open*.

eTable 2. Full Model Coefficients for Stunting, Underweight, and Overweight at First and Last Visits [a]

a. Stunting

Parameter	First visit OR (95% CI)	First visit P value	Last visit OR (95% CI)	Last visit P value
Family diet: vegan	1.11 (0.92, 1.34)	0.3	1.05 (0.71, 1.54)	0.82
Family diet: vegetarian	1.08 (0.98, 1.18)	0.11	1.07 (0.9, 1.26)	0.45
Vaginal birth	0.82 (0.8, 0.83)	<0.001	0.89 (0.85, 0.93)	<0.001
Gestational week	0.3 (0.3, 0.31)	<0.001	0.8 (0.79, 0.82)	<0.001
Male sex	1.41 (1.38, 1.44)	<0.001	1.68 (1.61, 1.75)	<0.001
Maternal age at birth	0.9 (0.89, 0.91)	<0.001	0.91 (0.89, 0.93)	<0.001
Parity: 2	0.92 (0.89, 0.94)	<0.001	1.03 (0.98, 1.09)	0.25
Parity: 3	0.88 (0.85, 0.91)	<0.001	1.09 (1.02, 1.16)	0.01
Parity: 4	1.01 (0.97, 1.06)	0.62	1.23 (1.13, 1.34)	<0.001
Parity: ≥5	1.11 (1.07, 1.16)	<0.001	1.59 (1.48, 1.72)	<0.001
Full nursing: 1–6 mo			1.23 (1.17, 1.29)	<0.001
Full nursing: ≥7 mo			2.04 (1.83, 2.27)	<0.001
Partial nursing: 1–6 mo			1.01 (0.94, 1.08)	0.83
Partial nursing: 7–11 mo			1.18 (1.09, 1.27)	<0.001
Partial nursing: ≥12 mo			1.54 (1.43, 1.66)	<0.001
Geographic-level income	0.91 (0.9, 0.92)	<0.001	0.79 (0.77, 0.81)	<0.001
Arab residence	0.8 (0.78, 0.82)	<0.001	0.29 (0.27, 0.31)	<0.001
Mixed residence	0.86 (0.84, 0.89)	<0.001	0.71 (0.67, 0.74)	<0.001

b. Underweight

Parameter	First visit OR (95% CI)	First visit P value	Last visit OR (95% CI)	Last visit P value
Family diet: vegan	1.37 (1.15, 1.63)	<0.001	1.06 (0.5, 2.23)	0.89
Family diet: vegetarian	1.21 (1.11, 1.32)	<0.001	0.8 (0.55, 1.15)	0.22
Vaginal birth	0.94 (0.92, 0.97)	<0.001	1.05 (0.97, 1.15)	0.25
Gestational week	0.81 (0.8, 0.82)	<0.001	0.87 (0.84, 0.9)	<0.001
Male sex	1.14 (1.12, 1.17)	<0.001	2.06 (1.9, 2.23)	<0.001
Maternal age at birth	1.02 (1.01, 1.03)	0.001	1.1 (1.06, 1.15)	<0.001
Parity: 2	0.89 (0.86, 0.92)	<0.001	0.9 (0.81, 1)	0.05
Parity: 3	0.9 (0.87, 0.94)	<0.001	0.96 (0.85, 1.08)	0.5
Parity: 4	0.89 (0.84, 0.94)	<0.001	0.89 (0.75, 1.05)	0.17

© 2026 Avital K et al. *JAMA Network Open*.

Parity: ≥5	1.09 (1.04, 1.15)	<0.001	0.84 (0.71, 1)	0.05
Full nursing: 1–6 mo			0.95 (0.87, 1.04)	0.28
Full nursing: ≥7 mo			1.21 (0.92, 1.58)	0.17
Partial nursing: 1–6 mo			1.05 (0.94, 1.17)	0.37
Partial nursing: 7–11 mo			0.75 (0.66, 0.87)	<0.001
Partial nursing: ≥12 mo			0.99 (0.87, 1.13)	0.88
Geographic-level income	0.87 (0.86, 0.88)	<0.001	0.91 (0.87, 0.96)	<0.001
Arab residence	0.61 (0.59, 0.63)	<0.001	0.79 (0.71, 0.87)	<0.001
Mixed residence	1.05 (1.02, 1.08)	0.002	1.05 (0.95, 1.16)	0.33

c. Overweight

Parameter	First visit OR (95% CI)	First visit P value	Last visit OR (95% CI)	Last visit P value
Family diet: vegan	0.86 (0.61, 1.2)	0.37	0.91 (0.61, 1.35)	0.63
Family diet: vegetarian	0.93 (0.8, 1.07)	0.32	1.01 (0.86, 1.18)	0.89
Vaginal birth	0.85 (0.82, 0.88)	<0.001	0.82 (0.79, 0.85)	<0.001
Gestational week	1.02 (1, 1.04)	0.01	1.02 (1, 1.04)	0.03
Male sex	1.49 (1.44, 1.54)	<0.001	1.11 (1.07, 1.15)	<0.001
Maternal age at birth	0.98 (0.96, 1)	0.01	1.01 (0.99, 1.03)	0.35
Parity: 2	1.15 (1.1, 1.2)	<0.001	0.98 (0.93, 1.03)	0.39
Parity: 3	1.2 (1.14, 1.26)	<0.001	1.04 (0.98, 1.1)	0.15
Parity: 4	1.2 (1.12, 1.28)	<0.001	0.98 (0.9, 1.06)	0.55
Parity: ≥5	1.22 (1.15, 1.3)	<0.001	1 (0.92, 1.09)	>0.9
Full nursing: 1–6 mo			0.78 (0.75, 0.82)	<0.001
Full nursing: ≥7 mo			0.85 (0.73, 0.99)	0.04
Partial nursing: 1–6 mo			0.96 (0.91, 1.01)	0.1
Partial nursing: 7–11 mo			0.86 (0.81, 0.91)	<0.001
Partial nursing: ≥12 mo			0.73 (0.69, 0.78)	<0.001
Geographic-level income	0.92 (0.91, 0.94)	<0.001	1.05 (1.03, 1.07)	<0.001
Arab residence	1.17 (1.12, 1.22)	<0.001	0.99 (0.95, 1.04)	0.73
Mixed residence	0.94 (0.9, 0.98)	0.004	0.94 (0.89, 0.99)	0.01

[a] First visit is defined as birth (age = 0), while initial measurements for length were obtained within the first 60 days of life. The last visit for length and weight was between 700 and 810 days of age (~2 years).

Model adjusted for maternal age, gestational week, birth type, and parity geographic-level income measure, and area-level ethnic composition. For last visit outcomes, the model was also adjusted for breastfeeding categories (full and partial)duration.

Outcome definitions (based on WHO standards): Underweight: weight-for-length z-score < −2; Overweight: weight-for-length z-score > +2; Stunting: length-for-age z-score < −2

© 2026 Avital K et al. *JAMA Network Open*.

eTable 4. Sensitivity Analysis of Associations Between Family Dietary Pattern and Infant Length and Length-for-Age *z* Scores in Subgroups Defined by Measurement Sufficiency

a. Infants With Sufficient Growth Measurements[a] (N=561,790)

Outcome		Model 1		Model 2		Model 3	
	Family diet	Beta (95% CI)	P Value	Beta (95% CI)	P Value	Beta (95% CI)	P Value
Length	Omnivore	Ref	-	Ref	-	Ref	-
	Vegan	-0.23 (-0.35, -0.1)	<0.001	-0.21 (-0.35, -0.06)	0.005	0.09 (-0.03, 0.22)	0.16
	Vegetarian	0 (-0.06, 0.05)	>0.9	-0.01 (-0.07, 0.05)	0.71	0.06 (0.01, 0.12)	0.02
LAZ	Omnivore	Ref	-	Ref	-	Ref	-
	Vegan	-0.08 (-0.14, -0.03)	0.002	-0.09 (-0.15, -0.03)	0.005	0.04 (-0.01, 0.09)	0.1
	Vegetarian	0 (-0.02, 0.03)	0.79	0 (-0.03, 0.02)	0.81	0.03 (0.01, 0.05)	0.007

b. Infants With Limited Growth Measurements[a] (N= 637,028)

		Model 1		Model 2		Model 3	
Outcome	Family diet	Beta (95% CI)	P Value	Beta (95% CI)	P Value	Beta (95% CI)	P Value
Length (cm)	Omnivore	Ref	–	Ref	–	Ref	–
	Vegan	-0.11 (-0.2, -0.01)	0.03	-0.16 (-0.27, -0.06)	0.002	0.14 (0.05, 0.23)	0.002
	Vegetarian	0.10 (0.05, 0.14)	p<0.001	0.00 (-0.05, 0.05)	p>0.9	0.12 (0.07, 0.17)	p<0.001
LAZ	Omnivore	Ref	-	Ref	-	Ref	-
	Vegan	-0.05 (-0.09, -0.01)	0.02	-0.08 (-0.12, -0.03)	p<0.001	0.06 (0.02, 0.10)	0.002
	Vegetarian	0.04 (0.02, 0.06)	p<0.001	0.00 (-0.02, 0.02)	0.87	0.05 (0.03, 0.07)	p<0.001

Abbreviations: LAZ, length-for-age z score; Ref, reference group (omnivores); CI: Confidence interval

The mixed-effects models employed five knots for age. Model 1 was adjusted for the child age and sex. Model 2 included additional adjustments for maternal age, gestational week, birth type, parity, and nursing status, geographic-level income measure, and area-level ethnic composition. Model 3 incorporated further adjustments for birth weight.

© 2026 Avital K et al. *JAMA Network Open*.

eTable 5. Sensitivity Analysis of Associations Between Family Dietary Patterns and Growth Outcomes in Subgroups Defined by Measurement Sufficiency [a]

a. Infants With Sufficient Growth Measurements[a] (N=561,790)

Outcome [b]	Visit [c]	Family Diet	Prevalence, n (%)	Model 1 [d]		Model 2 [e]	
				OR (95% CI)	P Value	OR (95% CI)	P Value
**Stunting**	**First visit**	**Omnivore**	36,719 (7.3)	Ref	–	Ref	–
		**Vegetarian**	453 (7.2)	1.01 (0.92, 1.11)	0.86	1.02 (0.9, 1.16)	0.76
		**Vegan**	75 (6.6)	0.93 (0.73, 1.17)	0.52	1.12 (0.83, 1.51)	0.46
	**Last visit**	**Omnivore**	15,512 (3.1)	Ref	–	Ref	–
		**Vegetarian**	209 (3.3)	1.1 (0.95, 1.26)	0.2	1.11 (0.94, 1.3)	0.23
		**Vegan**	43 (3.8)	1.26 (0.93, 1.7)	0.14	1.09 (0.75, 1.6)	0.64
**Underweight**	**First visit**	**Omnivore**	23,603 (4.7)	Ref	–	Ref	–
		**Vegetarian**	335 (5.4)	1.14 (1.02, 1.27)	0.02	1.16 (1.02, 1.32)	0.03
		**Vegan**	77 (6.8)	1.46 (1.16, 1.84)	0.002	1.38 (1.03, 1.84)	0.03
	**Last visit**	**Omnivore**	4,078 (0.8)	Ref	–	Ref	–
		**Vegetarian**	41 (0.7)	0.81 (0.6, 1.11)	0.19	0.85 (0.6, 1.2)	0.36
		**Vegan**	12 (1.1)	1.33 (0.75, 2.36)	0.32	1.17 (0.58, 2.35)	0.66
**Overweight**	**First visit**	**Omnivore**	11,897 (2.4)	Ref	–	Ref	–
		**Vegetarian**	122 (2.0)	0.84 (0.7, 1.01)	0.06	0.87 (0.7, 1.09)	0.22
		**Vegan**	18 (1.6)	0.69 (0.43, 1.11)	0.12	0.66 (0.36, 1.2)	0.17
	**Last visit**	**Omnivore**	19,599 (3.9)	Ref	–	Ref	–
		**Vegetarian**	231 (3.7)	0.95 (0.83, 1.09)	0.46	1.02 (0.87, 1.19)	0.81
		**Vegan**	39 (3.4)	0.89 (0.64, 1.22)	0.46	0.96 (0.66, 1.4)	0.85

b. Infants With Limited Growth Measurements (N= 637,028) outcomes for first visit only [a]

Outcome [b]	Family Diet	Prevalence, n (%)	Model 1 [d]		Model 2 [e]	
			OR (95% CI)	P Value	OR (95% CI)	P Value
**Stunting**	Omnivore	35,842 (6.9)	Ref	–	Ref	–
	vegetarian	473 (6.7)	1 (0.91, 1.1)	p>0.9	1.16 (1.03, 1.3)	0.01
	vegan	132 (7.2)	1.09 (0.92, 1.31)	0.32	1.19 (0.94, 1.5)	0.15

© 2026 Avital K et al. *JAMA Network Open*.

**Underweight**	Omnivore	24,637 (4.7)	Ref	–	Ref	–
	vegetarian	421 (6.0)	1.23 (1.12, 1.36)	p<0.001	1.24 (1.1, 1.39)	p<0.001
	vegan	137 (7.5)	1.52 (1.27, 1.81)	p<0.001	1.5 (1.22, 1.84)	p<0.001
**Overweight**	Omnivore	12,294 (2.4)	Ref	–	Ref	–
	vegetarian	140 (2.0)	0.87 (0.74, 1.03)	0.12	1.01 (0.84, 1.22)	0.89
	vegan	35 (1.9)	0.86 (0.62, 1.21)	0.39	0.99 (0.67, 1.45)	p>0.9

[a] Sufficient visits defined as ≥3 length measurements including ≥1 near 24 months; Limited visits defined as <3 length measurements or none near 24 months

[b] Outcome definitions (based on WHO standards): Underweight: weight-for-length z-score < −2; Overweight: weight-for-length z-score > +2; Stunting: length-for-age z-score < −2

[c] First visit is defined as birth (age = 0), while initial measurements for length were obtained within the first 60 days of life. The last visit for length and weight was between 700 and 810 days of age (~2 years).

[d] Model 1 was adjusted for sex and age.

[e] Model 2 was additionally adjusted for maternal age, gestational week, birth type, and parity geographic-level income measure, and area-level ethnic composition. For last visit outcomes, Model 2 was also adjusted for breastfeeding categories.

© 2026 Avital K et al. *JAMA Network Open*.

eTable 6. Baseline Characteristics by Follow-Up Completeness: Sufficient vs Limited Length Measurements

Characteristic	Sufficient visits N = 561,790[a, b] No. (%)	Limited visits N = 637,028[a, b] No. (%)	p-value[2]
**Family Nutrition**			<0.001
**Omnivore**	553,826 (99%)	626,864 (98%)	
**Vegan**	1,238 (0.2%)	2,100 (0.3%)	
**Vegetarian**	6,726 (1.2%)	8,064 (1.3%)	
**Maternal characteristics**			
**Mother Birth Age**	29.8 (5.6)	30.2 (5.7)	<0.001
**GLI** [c] Mean (SD)	4.38 (2.53)	4.39 (2.70)	0.004
**Area-level ethnic composition** [d]			<0.001
**Jewish**	278,415 (56%)	387,627 (67%)	
**Mixed**	100,257 (20%)	113,655 (20%)	
**Arab**	122,456 (24%)	75,155 (13%)	
**Parity**	1.89 (1.55)	1.91 (1.74)	<0.001
**Full Nursing Cat**			<0.001
**None**	271,511 (48%)	293,429 (46%)	
**1-6m**	279,587 (50%)	322,561 (51%)	
**7m or more**	10,621 (1.9%)	20,934 (3.3%)	
**Partial Nursing Cat**			<0.001
**None**	90,705 (16%)	112,112 (18%)	
**1-6m**	226,915 (40%)	240,113 (38%)	
**7m or more**	244,099 (43%)	284,699 (45%)	
**Birth outcomes**			
**Female**	259,151 (46%)	301,550 (47%)	
**Vaginal**	431,319 (77%)	502,756 (79%)	<0.001
**Gestation Week** Mean (SD)	39.13 (1.48)	39.22 (1.49)	<0.001
**Birth Weight** Mean (SD)	3.26 (0.46)	3.28 (0.46)	<0.001
**BWC** Mean (SD)	54 (28)	55 (28)	<0.001
**LBW**	26,984 (4.8%)	28,117 (4.4%)	<0.001
**SGA**	29,709 (6.8%)	35,105 (6.5%)	<0.001
**HBW**	27,663 (4.9%)	33,962 (5.3%)	<0.001
**LGA**	50,247 (12%)	63,132 (12%)	<0.001

[a]Pearson's Chi-squared test; One-way t-test analysis of means (not assuming equal variances).
[b] Sufficient visits defined as ≥3 length measurements including ≥1 near 24 months; Limited visits defined as <3 length measurements or none near 24 months.
[c]GLI is an area-based income index (0–10) derived from the child's residential geographic statistical area; higher values indicate higher neighborhood income.
[d] Defined by municipality population: Jewish >90% Jewish, Mixed >10%–<90%, Arab >90% Arab; reflects area, not individual ethnicity.
Abbreviations: GLI, Geographic-level income; BWC, Birth weight centile for gestational week; LBW, Low birth weight (<2.5 kg); SGA, Small for gestational age (<10 BW centile); HBW: High birth weight (>4kg); LGA, Large for gestational age (>90 BW centile); SD, Standard deviation;

© 2026 Avital K et al. *JAMA Network Open*.
