# Supplementary material for: Growth Trajectories in Infants From Families With Plant-Based or Omnivorous Dietary Patterns
Source: JAMA Netw Open. 2026 Feb 5;9(2):e2557798. doi: 10.1001/jamanetworkopen.2025.57798 (PMC12878416; doi:10.1001/jamanetworkopen.2025.57798)
Supplement: Supplement 3. — Data Sharing Statement [file jamanetwopen-e2557798-s003.pdf]

Data Sharing Statement

Avital. Growth Trajectories in Infants From Families With Plant-Based or Omnivorous Dietary Patterns. *JAMA Netw Open*. Published February 05, 2026.
doi:10.1001/jamanetworkopen.2025.57798

Data
Data available: No

Additional Information
Explanation for why data not available: The data are not publicly available due to Israeli Ministry of Health regulations and participant confidentiality requirements.
